# Optimization of ZnO-NPs to Investigate Their Safe Application by Assessing Their Effect on Soil Nematode *Caenorhabditis elegans*

**DOI:** 10.1186/s11671-015-1010-4

**Published:** 2015-07-28

**Authors:** Shruti Gupta, Tanuja Kushwah, Ashutosh Vishwakarma, Shweta Yadav

**Affiliations:** Department of Zoology, School of Biological Sciences, Dr H S Gour Central University, Sagar, 470003 MP India

**Keywords:** *C.elegans*, ZnO-NPs, Nanotoxicity, Biodistribution, *sod-1*, *mtl-1*, Nanouptake

## Abstract

Zinc oxide nanoparticles (ZnO-NPs) are increasingly receiving attention due to their widespread application in cosmetics, pigments and coatings. This has raised concerns in the public and scientific communities regarding their unexpected health effects. Toxicity effect of ZnO-NPs on the environment was assessed in the present study using *Caenorhabditis elegans*. Multiple toxicity end points including their mortality, behaviour, reproduction, in vitro distribution and expression of stress response *mtl-1* and *sod-1* genes were observed to evaluate safe application of ZnO-NPs. *C. elegans* were exposed to 10, 50, and 100 nm ZnO-NPs (0.1 to 2.0 g/l). Application of 10 nm ≥0.7g/l adversely affects the survivability of worms and was significantly not affected with exposure of 50 and 100 nm ≤1.0 g/l. However, reproduction was affected at much low concentration as compared to their survivability. LC50 was recorded 1.0 ± 0.06 (g/l) for 100 nm, 0.90 ± 0.60 for 50 nm and 0.620 ± 0.08 for 10 nm. Expression of *mtl-1* and *sod-1* was significantly increased with application of 10 nm ≥0.7g/l and significantly unaffected with exposure of 50 and 100 nm at the same concentration. ZnO-NPs (10 nm) had shown even distribution extended nearly the entire length of the body. The distribution pattern of ZnO-NPs indicates that the intestine is the major target tissues for NP toxicity. Study demonstrates that small-sized (10 nm) ZnO-NPs ≥0.7g/l is more toxic than larger-sized particles. This may be suggested on the basis of available data; application of 50 and 100 nm ≤1.0 g/l ZnO-NPs may be used to the environment as this shows no significant toxicity. However, further calibration is warranted to explore safe dose on soil compartments prior to their field application.

## Background

Nanotechnology is rapidly expanding the field of science with continuous development of nanomaterials and their industrial application. Nanoforms of metals, metal oxides, carbon-based materials and biopolymers are being used in several industries including diagnosis, drug delivery, cosmetics, sunscreens, food, paints, electronics, sports, imaging, etc. Among them, zinc oxide nanoparticles (ZnO-NPs) are most exploited at nano-dimension level. They are abundantly used nanomaterials in cosmetics and sunscreens as they exhibit high catalytic efficiency, as well as strong absorption ability for UV light. This makes them transparent and more aesthetically acceptable compared to their bulk counterpart as suggested by Schilling et al. [1]. ZnO-NPs have vast area of application including biosensor, gas sensor, cosmetics, storage, optical devices, window materials and drug delivery. Colloidal solutions of ZnO-NPs as nano-fertilizer (500–1000 ppm) have potential to boost yield and growth of crops. Prasad et al. [2] recorded application of ZnO-NPs (25 nm @ 1000 ppm) enhanced seed germination, seedling vigor, plant and root growth in peanuts. They are also being used in the food industry as additives and packaging due to their antimicrobial properties [3, 4]. Hu et al. [5] explored their potential use as fungicides in agriculture. Toxicological studies with NPs have mainly performed on aquatic organisms as recorded by Heinlaan et al. and Zhu et al. [6, 7]. However, Peralta-Videa et al. [8], Roh et al. [9], Hu et al. [5], Van der Ploeg et al. [10] and Gupta et al. [11] studied effects of short-term NP exposure to the isopod *Porcellio scaber*, *Caenorhabditis elegans* and earthworms *Eisenia fetida* and *Lumbricus rubellus*, respectively. Furthermore, researches are needed to provide insight into the toxicological effect of exposure to NPs on organisms especially living in the soil. Auffan et al. [12] reported metal-based NPs like ZnO, TiO_2_, Ag and CeO_2_; toxicity is at least partly due to the specific properties related to the small size and consequent high surface activity of NPs, while their effects may be further enhanced by the release of the free metal ions. If the free ions are more toxic than the original particles, this process of dissolution is likely to lead an increase of the overall toxicity. Speciation of metal NPs is soils is not yet clearly understood. Some works talk about the distribution of Zn, from ZnO-NPs in soil is the most liable fractions (those accessible to inhabiting soil organism) and differences between ZnO-NPs and derivate ion Zn^2+^ [13]. However, assessment of toxicity of the ionic forms of metal oxide nanoparticles may require particular attention on their solubility. ZnO-NPs can impose serious toxicity to bacteria, *Daphnia magna*, freshwater microalgae, mice and even human cell [14–17]. These nanoparticles have the ability to penetrate to the skin and to exert toxicity to viable cells. Jeng and Swanson [18] observed dramatic changes in cell morphology and apoptosis in mammalian cell after exposure of 50 μg/ml for 24 h. At exposure of 100 μg/ml, the recorded death of the cells was 15 to 50 %; Jeng and Swanson [18] also observed reduced mitochondrial activity (>80%).. Mitochondrial function showed that ZnO exhibits more toxicity than other metal oxide nanoparticles [19]. Jeng and Swanson [18] also observed reduced mitochondrial activity (>80 %).

The increased production and use of ZnO-NPs enhances the probability of their exposure in occupational and environmental settings. Although this NP has great commercial importance and present in various commercial products, so therefore is growing concern in public and scientific communities regarding their unanticipated and adverse health effects. A significant portion of ZnO-NPs may be expected to be released in sewage sludge via waste water. Varying portions of sewage sludge were disposed in landfills and incinerated or applied to agricultural land [20]. Therefore, terrestrial ecosystems are expected to be an ultimate sink for a large portion of NPs. This raises concerns about potential effects, entry in food webs and ultimately human exposure from consumption of contaminated agricultural products. In the current study, the toxicity of different-sized ZnO-NPs were assessed on the soil nematodes *C. elegans* using multiple toxicity end points to record optimal dose and size of these NPs for their safe application*.*

## Methods

### Test Compounds

ZnO-NPs (100, 50 and 10 nm) were purchased from Sigma Aldrich Chemical (St Louis, MO, USA). Particles were labelled as suggested by Tachikawa et al. [21] with fluorescent polymer. The size of the particles was measured in 20-μl particle suspension from the test medium on 400 mesh carbon-coated copper grid and observed using a transmission electron microscope (40-100KV) at Sophisticated Analytical Instrumentation Facility of Electron Microscopy, Department of Anatomy, All India Institute of Medical Sciences, New Delhi, India.

### Test Organism and Method of Exposure

The wild-type *C. elegans* Bristol strain N2 was obtained from Caenorhabditis Genetic Centre (CGC), USA, and culture was maintained on nematode growth medium (NGM) plates seeded with *Escherichia coli* strain OP50 at 20 °C, using the standard method [22]. Young adult (3 days old) synchronized culture were used in all the experiments. Worms were incubated at 20 °C for 24 h without a food source and were then subjected to the analysis [9]. Nematodes were exposed to three different-sized ZnO-NPs (10, 50 and 100nm). The test consisted series of seven ZnO-NP concentrations (0.1–2.0 g/l). NPs were diluted in K-medium (32 mM KCl, 51 mM NaCl) following Williams and Dusenbery [23] and buffered in 140 mM sodium acetate (pH 6.0) to avoid aggregation. Each treatment was replicated for three times, and control (K-medium + buffer) was maintained for the entire test.

### Assessment of Mortality and Behaviour

Toxicity end points including LC50 (probit method), mortality and mobility were observed after 24-h incubation with exposure of NPs, and juveniles were recorded at 48 h. Visually dead worms were sorted out with a platinum wire under stereo microscope.

### Analysis Expression of Stress Response Gene *mtl-1* and *sod-1*

After 24-h incubation with exposure of NPs, nematodes were harvested for the analysis of *mtl-1 (metal response proteins) and sod-1(anti oxidant enzyme)* gene expression. Standard procedures were followed as suggested by Roh et al. [24]. Reverse transcription (two steps) polymerase chain reaction (RT-PCR) method was used, and PCR products were separated on 1.5 % agarose gel through electrophoresis and observed by using ethidium bromide. Relative density of each band was observed in BioRed gel documentation system.

### Assessment of In Vivo Distribution of NPs

After exposure of ZnO-NPs, fluorescence distribution images were observed by using fluorescence microscope equipped with a peltier cooled charge-coupled camera. Both differential interference contrast (DIC) and epi-fluorescence images were taken. Filter set with maxima of 460 nm was used for visualization of fluorescence.

### Statistical Analysis

Results are the means of three replicates. Two-way analysis of variance (ANOVA) was performed by using the SPSS 10.5 software. The objective of statistical analysis was to determine any significant differences among the parameters analysed in different treatments to record optimal dose and size of ZnO NPs for their safe application. *p* values used to decide statistical significance based on the somewhat arbitrary choice of level were often set at 0.05 except for mortality and behaviour (0.01).

## Results and Discussion

### Mortality and Behaviour of ZnO-NP-exposed *C. elegans*

**Fig. 1:**
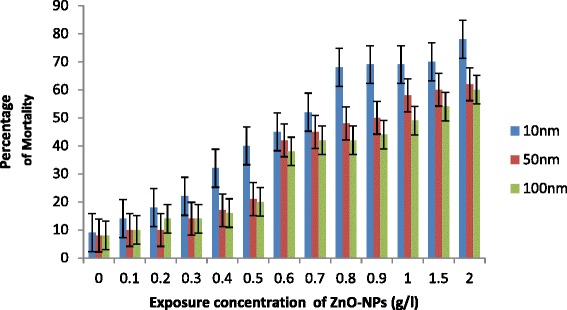
Percentage of mortality of *C. elegans* exposed to ZnO-NPs for 24 h. All the values are mean and standard deviation of three replicates (*p* < 0.01)

**Fig. 2:**
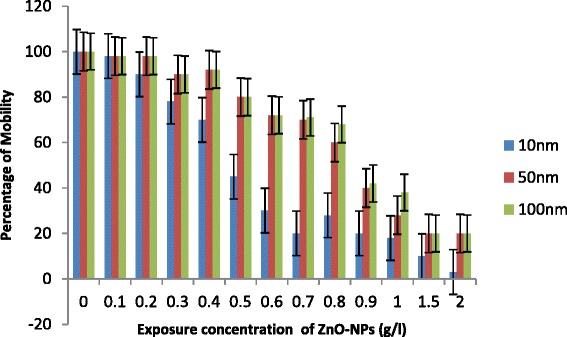
Percentage of mobility of *C. elegans* exposed to ZnO-NPs for 24 h. All the values are mean and standard deviation of three replicates (*p* < 0.01)

**Fig. 3:**
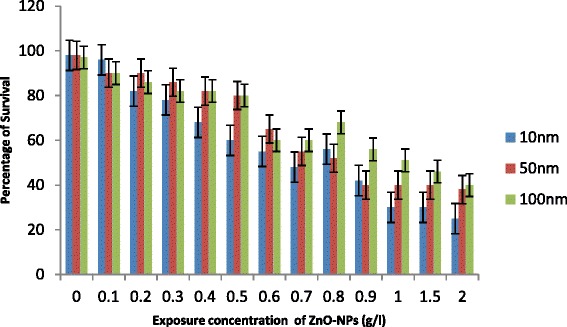
Percentage of survivability of *C. elegans* exposed to ZnO-NPs for 24 h. All the values are mean and standard deviation of three replicates (*p* < 0.01)

**Fig. 4:**
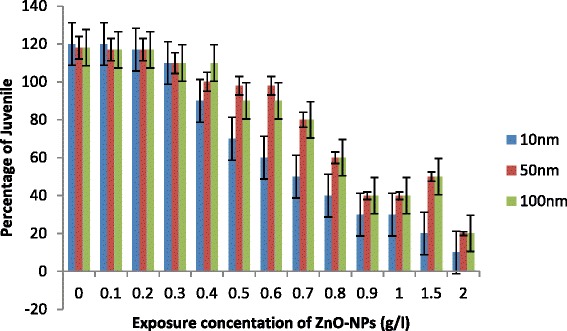
Percentage of number of juveniles of *C. elegans* exposed to ZnO-NPs for 24 h. All the values are mean and standard deviation of three replicates (*p* < 0.01)

Exposure of *C. elegans* to ZnO-NPs caused an increase in percentage of mortality with an increase in concentration (0.1 to 2.0g/l) and decrease in size of NPs (Fig. 1). Less than 10 % mortality was observed in control in all cases. LC50 was recorded 1.0 g/l for 100 nm ZnO NPs, 0.90 g/l for 50 nm ZnO-NPs and 0.62 g/l for 10 nm NPs (Fig. 1). Increase in percentage of mortality was not significantly important for 50 and 100 nm ≤1.0g/l, while 10 nm ZnO-NPs significantly increase the percentage of mortality even at ≥0.7 g/l. The first step to observe acute toxicity of any environmentally relevant species is to understand their possible adverse ecological effects of engineered nanoparticles released to the environment. Usually, metal ions inhibit the activity of important enzymes or disruption of the integrity of the cell membrane. Their small size, large surface and ability to generate reactive oxygen species appear to play an essential role for the nanoparticles to induce toxicity, as suggested by Oberdoster et al. [25] and Hu et al. [5]. Franklin et al. [16] suggested that the toxicity of ZnO-NPs may also be due to dissolution of Zn^2+^. However, bioavailability and toxicity of transition metals are controlled by large number of extrinsic factors including organic and inorganic ligands as suggested by Hyung et al. [26]. Free ionic forms of metals generally are considered to be more toxic to biota in comparison to precipitated, sorbed and complexed forms. In the present study, the apparently increased toxicity of sized (10 nm) ZnO-NPs (LC50, 0.62 g/l) may be attributed due to generation of more ROS as evident by enhanced expression of *sod-1* gene. However, increase in Zn^2+^ ion dissolution may not be ignored. Survival and behaviour movement of *C. elegans* with exposure of different sized NPs are shown in Figs. 1, 2 and 3. There were no significant differences that were recorded in percentage of mobility with exposure ≤1.0 g/l especially for 50 and 100 nm ZnO-NPs. In general, 10 nm ZnO-NPs adversely affect mortality (Fig. 1) and behaviour of worms (Fig. 2) at exposure ≥0.7g/l. A significant decrease in reproduction was observed (Fig. 4) with an increase in dose of ZnO-NPs and strongly affected with a decrease in size of NPs. Reproduction was affected even at low exposure (≥0.3g/l) compared to their rate of mortality and behaviour. Reduced fertility in present investigation may be due to compensatory mechanism to metabolize the toxicity induced by NPs as reported by Roh et al. [9] for other nanoparticles.

**Fig. 5:**
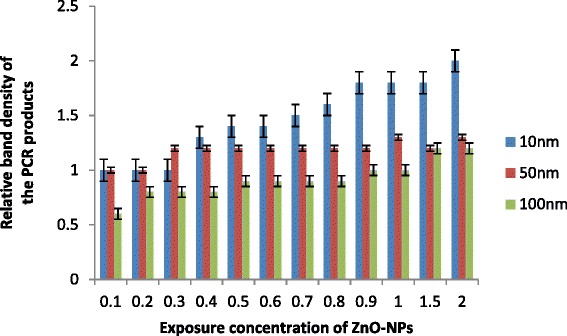
*sod-1* gene expression profiling in *C. elegans* exposed to ZnO-NPs for 24 h (standard error of the mean, *p* < 0.05)

### Expression of *mtl-1* and *sod-1* Gene

Gene expression may consider as high sensitivity and mechanistic values to diagnose environmental contamination. Nowadays, environmental stress response in toxicology was increasingly recorded by using gene profiling by many workers including Snell et al. [27], Lee and Choi [28], Roh et al. [24] and Poynton et al. [29]. In the present investigation, the effect of ZnO-NPs on alteration of gene expression after exposure of ZnO-NPs was analysed in two genes, namely, *mtl-1* and *sod-1* by using stress response gene expression profiling analysis [9]. Low-molecular weight cystine-rich protein, metallothionein (*mtl*), helps in metal detoxification and homeostasis. It is ubiquitous in most of the animals. A cell-specific factor, as well as developmentally modulated and metal response pathways controls their transcription. Due to their inducibility to transition metals, they are considered as an important specific biomarker to detect organism response to heavy metals. They have been involved in the defence against reactive oxygen species and also in the homeostatic regulation of heavy metals. Otuska [30] assumed them as multifunctional proteins with additional unidentified physiological roles. Therefore, *mtl-1* gene may serve as significant model for investigating the toxicity of ZnO-NPs and mechanism to cellular response of uptake of heavy metals.

**Fig. 6:**
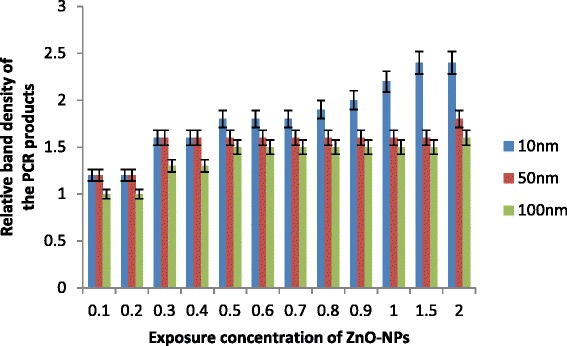
*mtl-1* gene expression profiling in *C. elegans* exposed to ZnO-NPs for 24 h. (standard error of the mean, *p* < 0.05)

Similarly, the making of superoxide dismutase is instructed by *sod-1* gene, which is abundantly in cells throughout the body. This enzyme attaches the molecules of zinc to break down toxic-charged oxygen molecules called superoxide radicals. There may be the possibility of high accumulation of harmful superoxide radicals with exposure of small-sized NPs (10 nm) in the present study. This may responsible for production of other types of toxic radicals including increased cell death or formation of aggregates of misfolded super oxide dismutase. No significant change was observed in expression of *mtl-1*gene for all ZnO-NPs in the present study. However, low expressions were recorded for 100 nm in all treatments (Fig. 5). This may be due to relatively high uptake of NPs and their accumulation by *C. elegans* for smaller-sized particles. Relative band density of PCR products for *mtl-1* enhanced for 10 nm at exposure of ≥0.5g/l and above that was most pronounced and significantly increased by 1.5 to 2.5. On the other hand, 50 and 100 nm band density almost remains constant. It appears that the exposure of 10 nm ZnO-NPs ≥0.4g/l increases the expression of *mtl-1* gene and that is responsible for uptake of Zn^+2^. Even, the expression of *mtl-1* gene was significantly increased in all treatments of 10 nm ZnO-NPs. Similarly, generation of reactive oxygen species was also significantly increased with an increase in concentration of 10 nm ZnO-NPs (Fig. 6). Data suggests that *mtl-1* and *sod-1* may be involved in the metabolism of ZnO-NPs and/or toxicity in *C. elegans*. Expression of both genes may consider as an early warning signal, relating a molecular level response to determine environmental stress of ZnO-NPs and their ecological effects. This also represents a substantial challenge to correlate validated toxicity end points including induced gene expression. In general, ZnO-NP toxicity may results from two more replacement of essential metals at protein active sites in 10 nm ZnO-NPs and may be due to the generation of reactive oxygen species at large for smaller-sized particles that damage macro-molecules, including DNA and proteins and thus may impair cellular function.

**Fig. 7:**
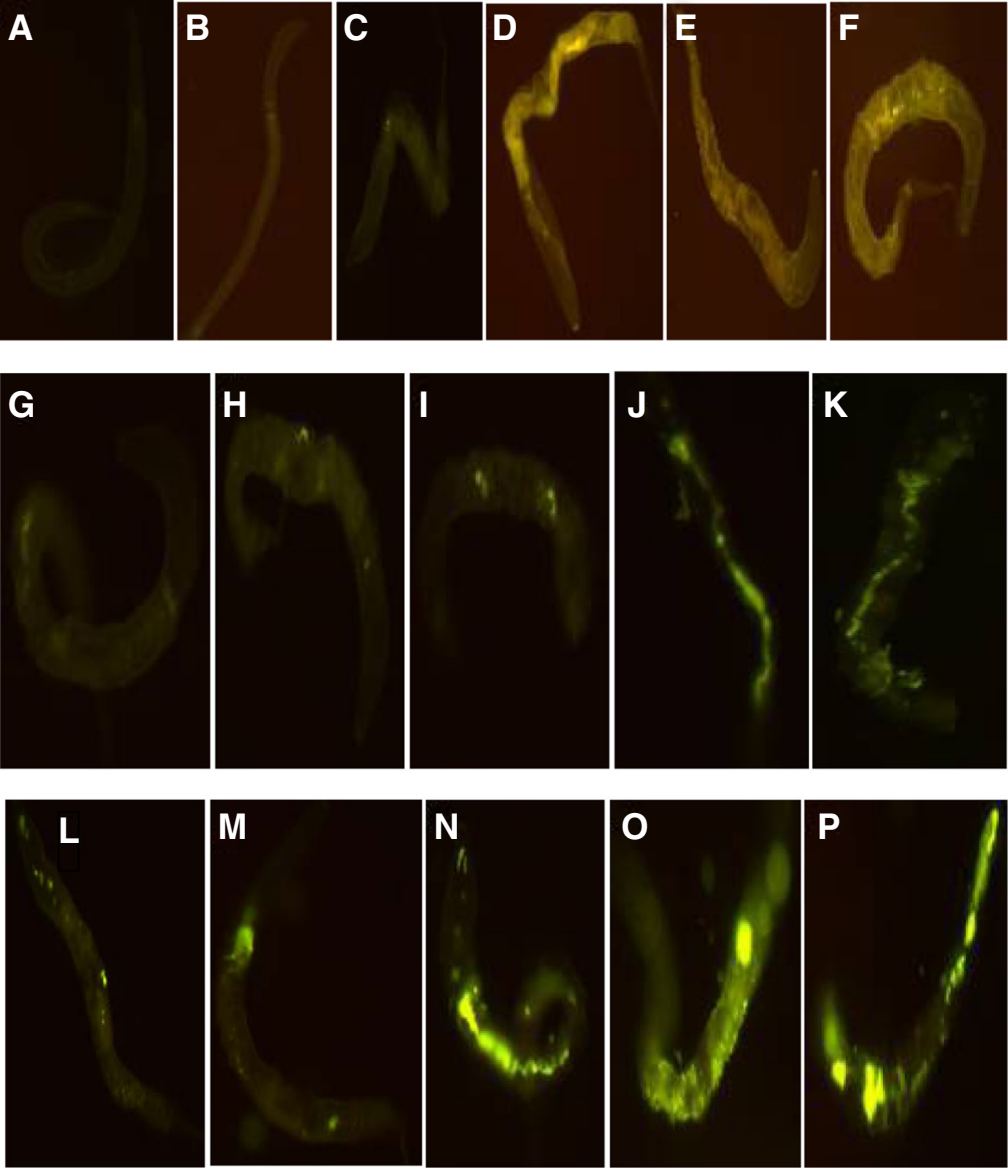
Zinc distribution images (×40) and their accumulation in *C. elegans* exposed to ZnO-NPs for 24 h. **a** control. **b** 0.2 g/l-100 nm. **c** 0.6 g/l-100 nm. **d** 1.0 g/l-100 nm. **e** 1.5 g/l-100 nm. **f** 2.0 g/l-100 nm. **g** 0.2 g/l-50 nm. **h** 0.6 g/l-50nm. **i** 1.0 g/l-50 nm. **j** 1.5 g/l-50 nm. **k** 2.0 g/l-50 nm. **l** 0.2 g/l-10 nm. **m** 0.6 g/l-10 nm. **n** 1.0 g/l-10 nm **o** 1.5 g/l-10m. **p** 2.0 g/l-10 nm

### Biodistribution of ZnO-NPs

**Fig. 8:**
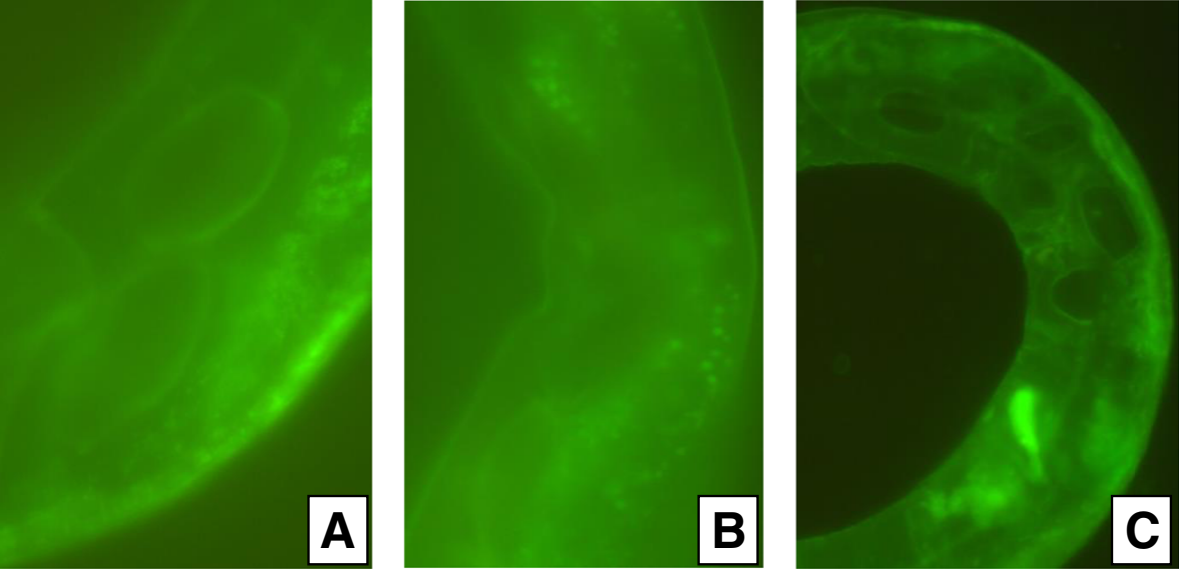
Zinc distribution images (×100) and their accumulation in eggs of *C. elegans* exposed to ZnO-NPs. **a** 50 nm. **b** 100 nm. **c** 10nm

Remarkable increase in fluorescence was observed in all treatments compared to controls. Fluorescence distribution images of *C. elegans* after exposure to ZnO-NPs showed high pixel intensities with increase in concentration and decrease in size of NPs. The highest pixel intensity was recorded with exposure of 10 nm NPs and shows more even distribution of fluorescence. However, at exposure of 100 nm (Fig. 7b–f) and 50 nm (Fig. 7g–k) ZnO-NPs, significant pixel fluorescence was recorded only in anterior region, while with 10 nm ZnO-NP-treated *C. elegans*, fluorescence extended nearly the entire length of the body(Fig. 7l–p). This may be due to high metal accumulation at exposure of 10 nm ZnO-NPs. It is known that accumulation of metals depends on physicochemical properties of metals and physiology of organisms. Essential and non-essential metals may also affect metal accumulation strategies. Zinc is an essential metal and plays an important role in diverse biological processes. However, the functional diversity of zinc shows sophisticated mechanism in regulation of its uptake and distribution within an organism [31]. The uniform distribution of fluorescence in 10 nm ZnO-NP-treated *C. elegans* confirmed the multiple biological functions in a wide range of cells and tissues by small nanoparticles, whereas an elevated intensity localized in particular area at exposure of 50 and 100 nm ZnO-NPs suggest the strategy that they accumulate NPs as “reservoir”. It stores extra Zn and releases it to support the organism’s need when Zn deficiency situation occurs as reported by Hongbo [32]. The distribution of ZnO-NPs in all the treatments was consistent up to 0.5 g/l within the gut rather than through the cuticle. At exposure of 50 and 100nm ZnO-NPs, fluorescence was recorded at particular points either in anterior or posterior end of the body with hotspots detected in the intestinal region. This might have induced toxicological effects in a number of cells and tissues at higher dose, which led to mortality. Even, significant difference in distribution pattern was not recorded at exposure of 50 and 100 nm. Data of kinetics of distribution in 50 and 100 nm ZnO-NP-treated *C. elegans* was almost similar. However, subtle difference was recorded at higher dose. The distribution pattern of ZnO-NPs may indicate that intestine is a major target tissue for NP toxicity. The fluorescence was also observed in eggs; a large number of eggs were recorded in 10-nm exposed worms (Fig. 8a–c). It is thought to be a strategy to enhance progeny survival and dispersal under stress. It appears that this tendency increased with exposure of high concentration and more prevalent in 10 nm ZnO-NPs. There may be chance of biotransformation of nanomaterials within *C. elegans* and observed fluorescence effects may be due to high rate of biotransformation of small-sized 10 nm NPs. The findings of the study may be helpful to determine the safe application of ZnO-NPs in the environment.

## Conclusions

The present study was focussed to optimize the safe application of ZnO-NPs by assessing multiple toxicity points and in vitro distribution in nanoparticle-exposed *C. elegans*. We conclude that the activity of worms was not adversely affected with exposure of 50 and 100 nm ZnO-NPs ≤1.0 g/l, while small-sized particles 10 nm ≥0.7g/l affects them at large as shown by an increase in the expression of *mtl-1* and *sod-1* gene. Distributional pattern of ZnO-NPs reveals that the intestine is the major target tissues for NP toxicity. The application of 50 and 100 nm ZnO-NPs may be safe to the environment in comparison to 10 nm. However, further calibration and validation of identified dose are warranted to explore more dose-response toxicity assessment and their kinetics of in vivo distribution.
